# Bibliometric and visual analysis of digital healthcare in cancer pain management via mobile apps

**DOI:** 10.1097/MD.0000000000043603

**Published:** 2025-08-01

**Authors:** Qiu-Song Shen, Hou-Ming Kan, Rui-Yu Wang, Nai-Fa Li, Sha Wang, Xue-Qin Rong

**Affiliations:** a Xinxiang Central Hospital, Fourth Clinical College of Xinxiang Medical College, Key Laboratory of Diagnosis, Treatment and Nursing of Critical Illness in Xinxiang City, Xinxiang, China; b Medical Sciences Division, School of Pharmacy, Macau University of Science and Technology, Macau SAR, China; c The Seventh Affiliated Hospital, Sun Yat-sen University, Shenzhen, Guangdong Province, China; d Department of Pain Spinal Minimally Invasive Centre, Sanya Central Hospital (Hainan Third People’s Hospital), Hainan Province, China.

**Keywords:** bibliometric analysis, cancer, cancer-related pain, digital healthcare, mobile app, visual analysis

## Abstract

**Background:**

Cancer pain is a common symptom among cancer patients, and although opioid drugs are effective, they have side effects and abuse issues. In recent years, mobile apps have gradually been used as digital healthcare for cancer pain management. By visualizing and analyzing the literature on the control of cancer pain through mobile apps, we aim to understand the current research status and hot issues in this field and explore future directions for digital smart healthcare research.

**Methods:**

CiteSpace software was used to visually analyze 252 articles on mobile app-based cancer pain control indexed by the Web of Science core collection database from 1995 to 2024. Microsoft Excel 2021 and GraphPad were used to draw world maps to evaluate the number of national articles and generate trend charts for annual publications.

**Results:**

Visual analysis revealed that the number of publications has been increasing annually. In terms of the number of published articles, the top 3 countries are the United States, China, and Australia. The analysis of highly cited literature indicates that the main apps involved are Pain Buddy, mHealth, The Pain Guard app, The mHealth Pain Squad+, and STAR, which target adults, children, and adolescents. Keywords and citation analysis indicate that quality of life, pain, breast cancer, palliative care, and mobile health may be hotspots and future directions of cancer pain research in mobile apps.

**Conclusion:**

Digital healthcare via mobile apps provides intelligent assistance for treating cancer pain, which is conducive to developing intelligent and painless cancer treatment and management strategies.

## 1. Introduction

Cancer is currently one of the leading causes of death worldwide, with nearly 10.0 million people succumbing to the disease, according to statistics from the World Health Organization in 2020.^[1]^ The 5 most common cancers are breast cancer (in females), prostate cancer, lung and bronchial cancer, colorectal cancer, and melanoma of the skin.^[2]^ Approximately 55% of cancer patients who receive treatment and 66% of cancer patients who do not receive treatment have experienced varying degrees of pain. The causes of cancer-related pain are quite complex and mainly include overlapping nociceptive pain, inflammatory pain, and neuropathic pain. Among these types of pain, neuropathic pain is common among all cancers.^[3]^ Quantitative and visual analyses have demonstrated that stem cells possess mechanisms to regulate neuropathic pain.^[4]^ Additionally, there are various methods available for treating cancer-related pain.^[5]^ The most commonly used medications are currently opioid analgesics. Although they are effective in managing cancer pain, their side effects are also increasing.^[6]^ Due to the continuous rise of mobile intelligence worldwide in recent years, smart devices are being developed at an accelerated pace.^[7]^ After decades of technological innovation, people are increasingly recognizing the enormous potential offered by mobile app digital healthcare.^[8]^ The latest research indicates that machine learning models, particularly those employing neural language processing, can facilitate early treatment for patients experiencing uncontrolled cancer pain.^[9]^ A recent cross-sectional survey on the remote medical management of cancer pain suggests that telehealth solutions can effectively address this issue.^[10]^ Mobile apps are programs that integrate opioid drugs and psychological and behavioral interventions.^[11]^ These applications not only improve the physical and mental health of patients,^[12]^ but also enhance their overall quality of life.^[13]^ Therefore, mobile apps are anticipated to become an effective strategy for managing cancer pain.

Bibliometrics involves the application of mathematical and statistical methods to analyze published scientific literature, aiding researchers in accurately understanding research trends and emerging topics.^[14]^ CiteSpace, a powerful bibliometric tool, can convert the relationships between various pieces of literature into intuitive scientific knowledge graphs. This functionality assists users in uncovering historical research trends and forming preliminary insights into future research directions.^[15]^ CiteSpace is particularly favored by researchers for analyzing the current landscape and trending topics within their fields. In this study, we utilized CiteSpace software to visualize and analyze relevant research on mobile apps in cancer pain management, while also predicting future development trends in this area.

## 2. Data and methods

### 2.1. Search strategy

The primary author of this study selected all relevant research literature on the use of mobile applications (apps) for managing cancer pain from 1995 to 2024 using a meticulously designed search strategy.

### 2.2. Retrieving the database

We conducted a literature search using the Web of Science (WOS) Core Collection database, which includes the Science Citation Index Expanded, Social Science Citation Index, and Emerging Sources Citation Index, and selected research findings related to the use of mobile apps in cancer pain management.

### 2.3. Search strategy

This study utilized MeSH keywords (https://www.ncbi.nlm.nih.gov/mesh) with the main keywords “mobile app” and “cancer pain.” For the classification of cancer pain, we referred to the 11th edition of the International Classification of Diseases (https://icd.who.int/en). A more detailed description is provided. The time frame for the literature review is limited to 1995 to 2024, and the selected types of literature include original research reports and review articles. For specific search terms and details of the search strategy, please refer to Table 1.

**Table 1:** Specific retrieval strategies.

Step	Search strategy	Result
#1.	TS = (cancer pain) OR (Oncological Pains) OR (Tumor-Related Pain Pain) OR (Tumor-Related) OR (Tumor Related Pain) OR (Tumor-Related Pains) OR (Tumor-Associated Pain) OR (Tumor Associated Pain) OR (Tumor-Associated Pains) OR (Oncology Pain) OR (Oncology Pains) OR (Cancer-Related Pain) OR (Cancer Related Pain) OR (Cancer-Related Pains) OR (Neoplasm-Associated Pain) OR (Neoplasm Associated Pain) OR (Neoplasm-Associated Pains) OR (Oncological Pain) OR (Cancer-Associated Pain) OR (Cancer Associated Pain) OR (Cancer-Associated Pains) OR (Neoplasm Related Pain) OR (Neoplasm-Related Pains)	94, 559
#2.	TS = (Mobile Applications) OR (Mobile Application) OR (Mobile App) OR (Portable Electronic Apps) OR (Portable Electronic App) OR (Portable Electronic Applications) OR (Portable Electronic Application) OR (Portable Software Apps) OR (Portable Software App) OR (Portable Software Applications) OR (Portable Software Application) OR (Mobile Health) OR (Telehealth)	221, 533
#3.	#1AND #2	344

Searched on October 1, 2024. Search time range: January 1, 1995 to October 1, 2024.

TS = theme.

### 2.4. Filter criteria

We conducted a comprehensive full-text search and established clear inclusion criteria.

### 2.5. Inclusion criteria

After a preliminary review of the title and abstract, we selected all literature related to the application of mobile applications in cancer pain management. The included literature was restricted to original research articles and reviews; conference letters and abstracts were excluded.

### 2.6. Number of studies included

**Figure 1.**
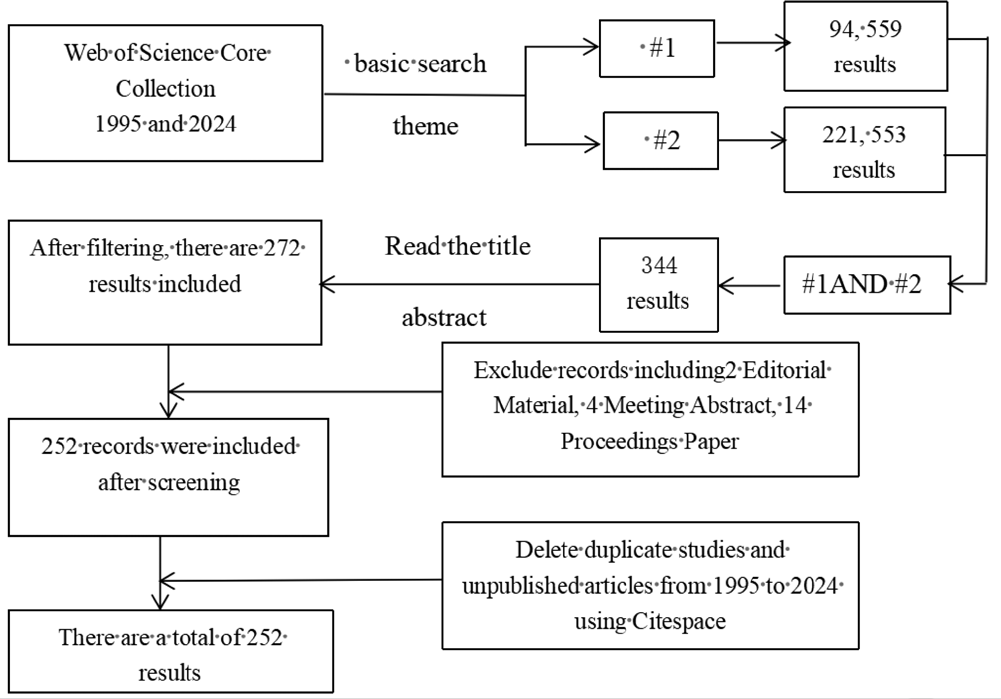
Process flowchart for article filtering.

On the basis of the established search keywords and strategies, we initially screened 344 records. By further reviewing the abstracts and excluding non-research content such as edited materials, letters, and conference abstracts, we ultimately identified 252 eligible records. The specific screening process is illustrated in Figure 1.

### 2.7. Research method

We conducted bibliometric and network visualization analyses on papers published from January 2007 to October 2024 using CiteSpace 6.3 R1 software (Drexel University, Philadelphia). This software encompasses various dimensions, including authors, journal co-citations, and keywords, with the aim of revealing research hotspots, core author groups, and journal influence. Additionally, we generated annual publication trend charts and world maps using Microsoft Excel 2021 to examine the development trends in this field from a macro perspective.

### 2.8. Bibliometrics and visualization analysis

Key elements, including publication year, institution, country, co-cited authors, references, and keywords, are comprehensively extracted from all publications. CiteSpace software was utilized for in-depth visualization processing to illustrate the complex relationships within the literature. This includes the correlation network, nodes, and their connection strength, centrality characteristics, and the formation of clusters.

## 3. Results

### 3.1. Publication statistics by year

**Figure 2.**
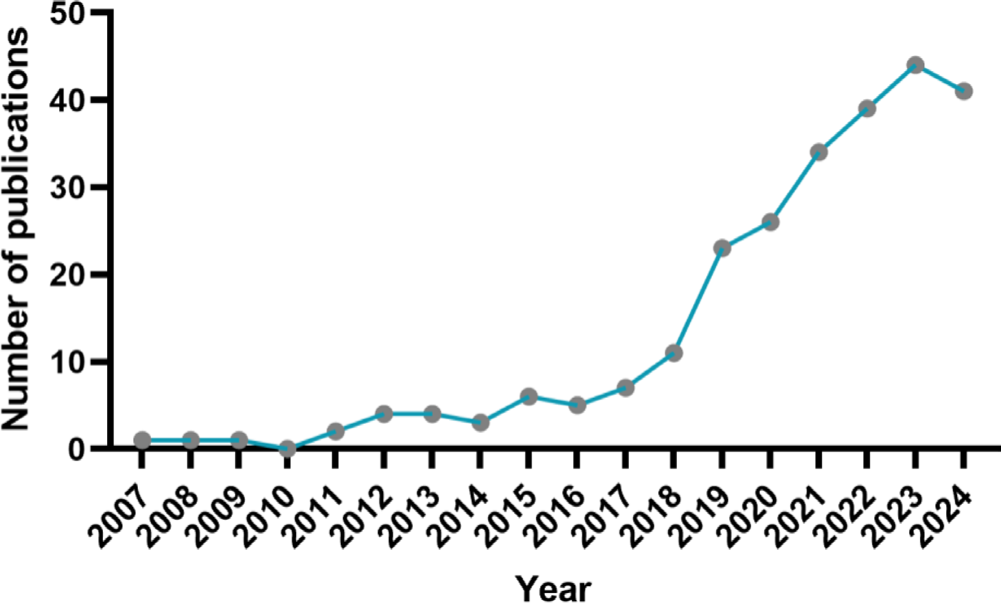
The bibliometric analysis results of publications produced.

This study included a total of 252 relevant publications. From 2007 to 2024, the volume of literature on the application of mobile app digital healthcare in the field of cancer pain management exhibited an annual increasing trend (as illustrated in Fig. 2). The year with the highest number of publications, totaling 44, is 2023, followed closely by 2024, which has 41 publications. Over the past 5 years, the number of publications in this field has surged to 184, representing 73% of the total publications. In terms of literature type, original research articles predominate, comprising 198 articles, or 78.6% of the total, while review articles account for 54 publications, representing 21.4%.

### 3.2. Author collaboration network analysis

**Figure 3.**
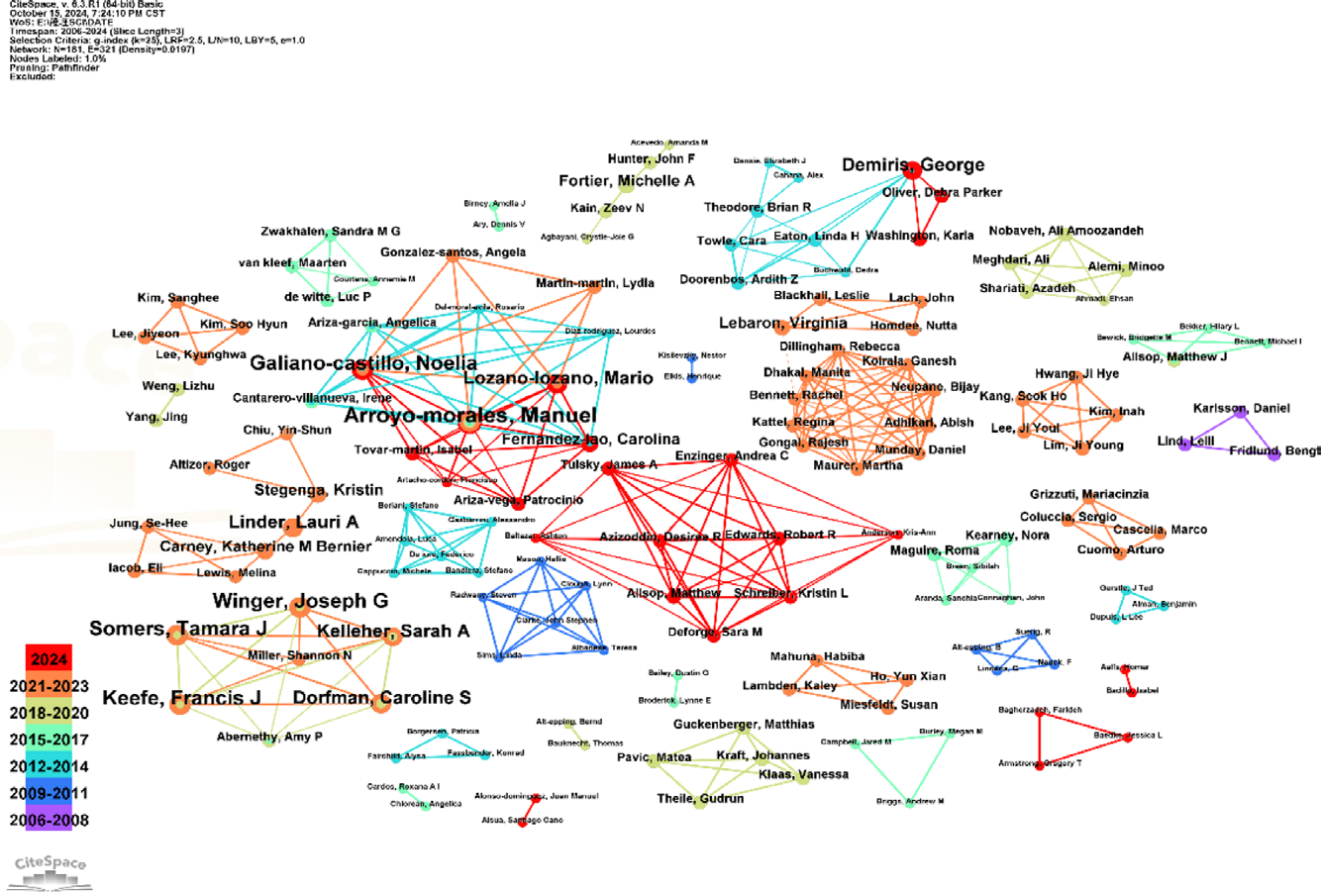
Analysis of author collaboration network.

Author collaboration network analysis reveals that the nodes in the graph are represented as annual ring graphs, with each band corresponding to an author’s publication volume for a given year. The greater the number of papers, the wider the corresponding annual ring nodes. Lines connecting the nodes indicate collaborative relationships between authors, with the color of the line representing the timing of the first collaboration; thicker lines denote a higher number of collaborations. The 5 authors with the highest publication counts are Arryo-Morels, Manuel, each with 6 articles, followed by Winger, Joseph G, Galiano-Castillo, Noelia, Somers, Tamara, and Keefe, Francis J, each with 5 articles. According to the color display, Arryo-Morales, Manuel, are highlighted in red, indicating recent publications. The specific author network analysis is illustrated in Figure 3.

### 3.3. Journal

**Figure 4.**
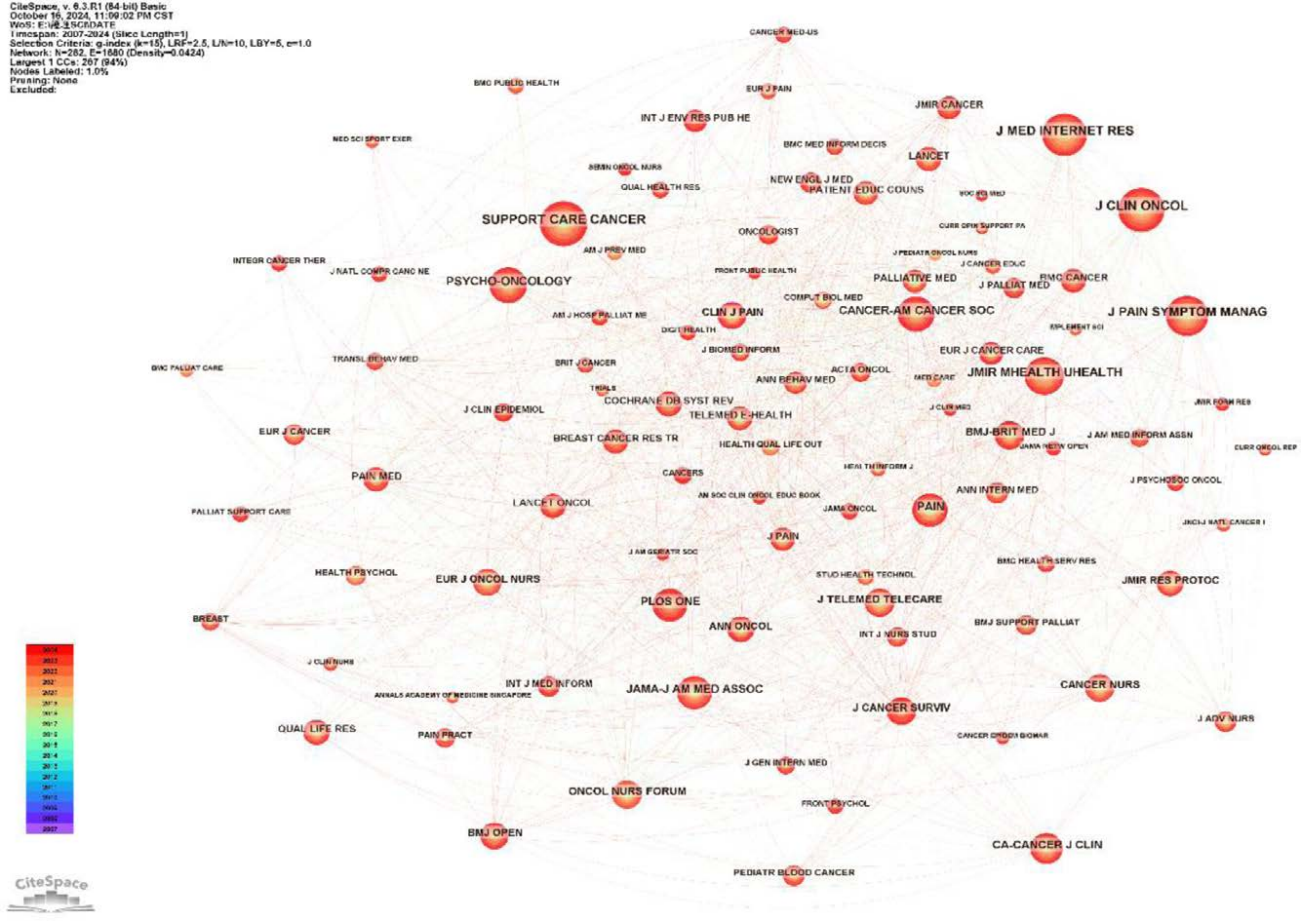
Analysis of journal co-citations.

Table 2 presents a comprehensive list of the top 15 co-cited journals. Among these, the journal with the highest citation count is “Support Care Cancer,” with 143 citations. Of the 15 journals, 10 are based in the United States, 2 in Canada, and 3 in the United Kingdom. The journal *Cancer-Am Cancer Soc* has the highest mediation centrality, with a centrality value of 0.19. By examining the level of centrality, one can identify the key core journals in this field. Additional details are illustrated in Figure 4.

**Table 2:** Journal co-citation information.

Rank	Co-citation frequency	Centrality	Citing journal	IF (2023)	National affiliation
1	143	0.06	*Support Care Cancer*	2.8	America
2	137	0.01	*J Clin Oncol*	42.1	America
3	126	0.04	*J Med Internet Res*	5.8	Canada
4	113	0.11	*J Pain Symptom Manag*	3.1	America
5	98	0.04	*JMIR Mhealth Uhealth*	5.3	Canada
6	89	0.06	*Psycho-Oncology*	3.3	Britain
7	86	0.19	*Cancer-Am Cancer Soc*	6.1	America
8	80	0.06	*Pain*	5.9	America
9	78	0.03	*JAMA-J Am Med Assoc*	63.1	America
10	77	0.04	*PLoS One*	2.8	America
11	70	0.07	*CA-Cancer J Clin*	503.1	America
12	57	0.09	*J Telemed Telecare*	3.5	Britain
13	57	0.12	*BMJ-Brit Med J*	93.6	Britain
14	57	0.06	*Oncol Nurs Forum*	1.6	America
15	54	0.06	*J Cancer Surviv*	3.1	America

### 3.4. Country analysis of coauthors

**Figure 5.**
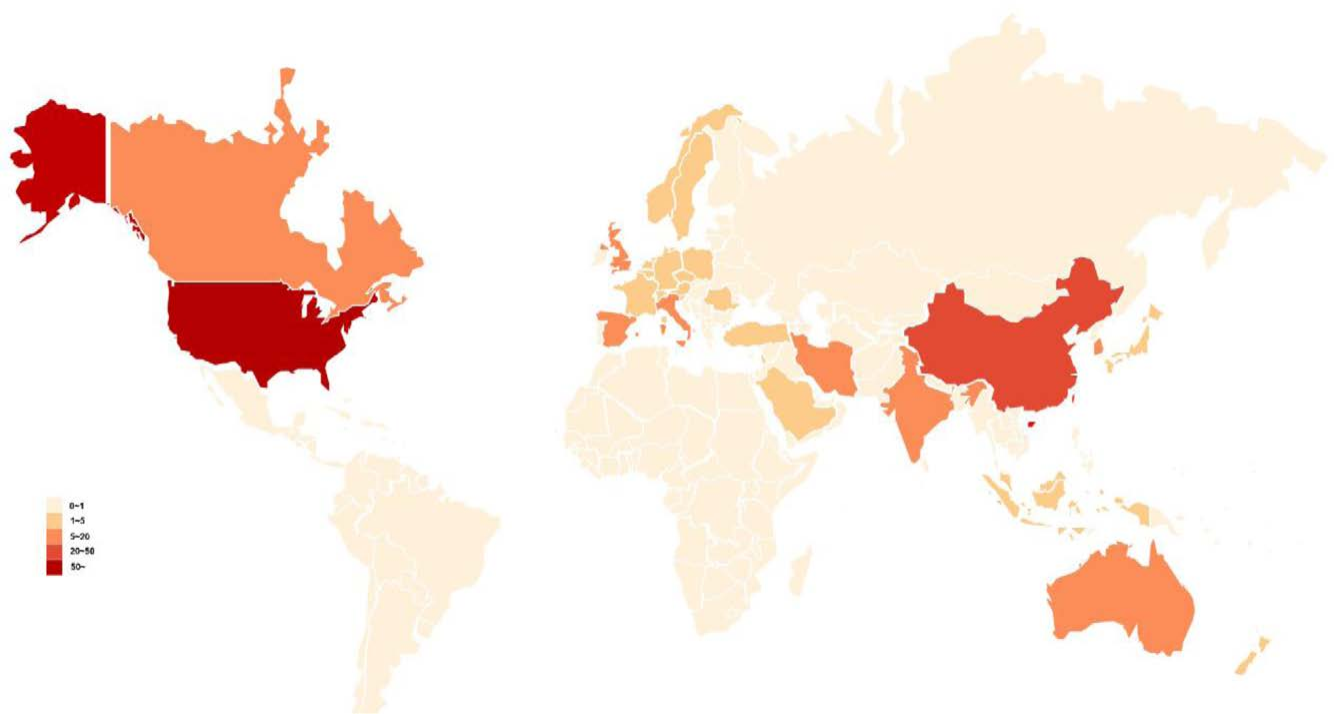
Country analysis of coauthors. The world map displays research on controlling cancer pain through mobile apps. There are more publications in the United States, China, and Australia.

On the basis of the WOS database, we utilized Microsoft Excel 2021 to create a world map illustrating the publication volume of each country. The results indicated that a total of 252 publications were produced by research teams from 32 countries. Among these, the United States led in publication volume, with 102 articles. The countries closely following the United States are China (n = 20), Australia (n = 16), and Spain (n = 16). In terms of citation rates, the United States surpasses other countries. Further details are presented in Figure 5.

### 3.5. Analysis of co-citations of literature

**Figure 6.**
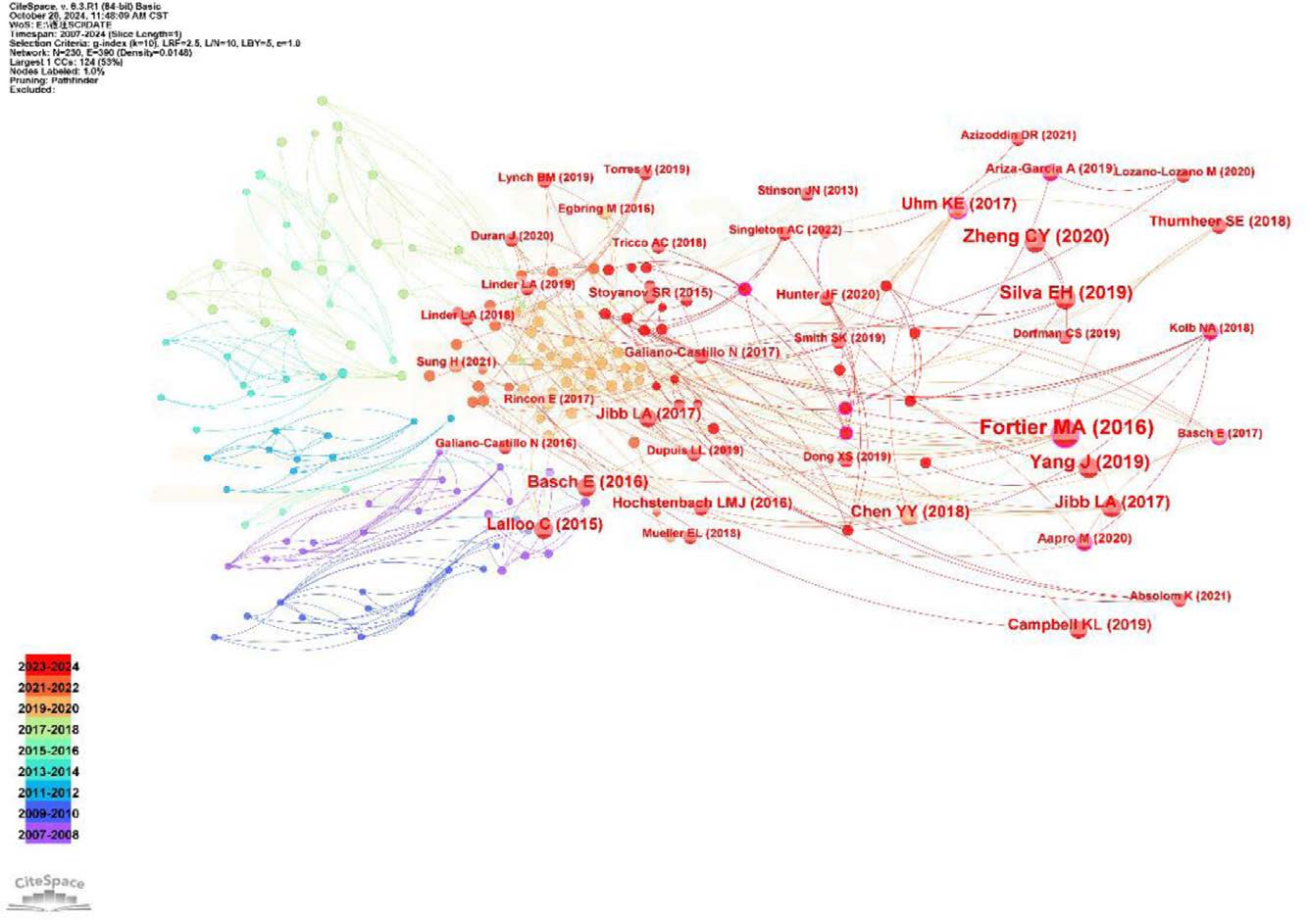
The co-citation analysis of the literature. Nodes and lines represent research literature and their co-citation relationships, with higher co-citation frequency indicating larger node sizes. The colors of the nodes and lines reflect the distance of the reference time, with warmer colors indicating closer times.

Table 3 shows the top 9 co-cited studies on controlling cancer pain through mobile apps.^[16–24]^ The most frequently cited studies are Fortier MA^[16]^ and 17citations. The literature with the highest centrality is Jibb LA^[19]^ (centrality = 0.37); the higher the centrality, the more frequently this literature is co-cited with other literature. Figure 6 shows the analysis of co-citations of literature. Figure 6 shows that the classic literature in this field has the largest node, and the literature with the largest node is Fortier MA^[16]^. The second article is Silva EH^[17]^. These 2 articles have received relatively high attention both domestically and internationally.

**Table 3:** The top 9 co-cited studies on controlling cancer pain through mobile apps.

Rank	Co-citation frequency	Centrality	First author (yr)	Research design type	Cancer population	App type
1	17	0.25	Fortier MA (2016)	Prospective descriptive research	Children	Pain Buddy
2	11	0.06	Silva EH (2019)	Systematic review	Unclassified	mHealth
3	11	0.00	Yang J (2019)	Experimental study	Adult	The Pain Guard app
4	9	0.02	Jibb LA (2017)	Prospective descriptive research	Adolescent	The mHealth Pain Squad+
5	8	0.06	Lalloo C (2015)	Prospective descriptive research	Children and adults	Smartphone applications
6	8	0.00	Basch E (2016)	Randomized controlled study	Adult	STAR
7	8	0.02	Zheng CY (2020)	Systematic review	Unclassified	Mobile apps
8	8	0.35	Chen YY (2018)	Meta-analysis	Unclassified	Telehealth intervention
9	7	0.37	Jibb LA (2017)	Prospective descriptive research	Adolescents	Smartphone app

### 3.6. Keyword

**Figure 7.**
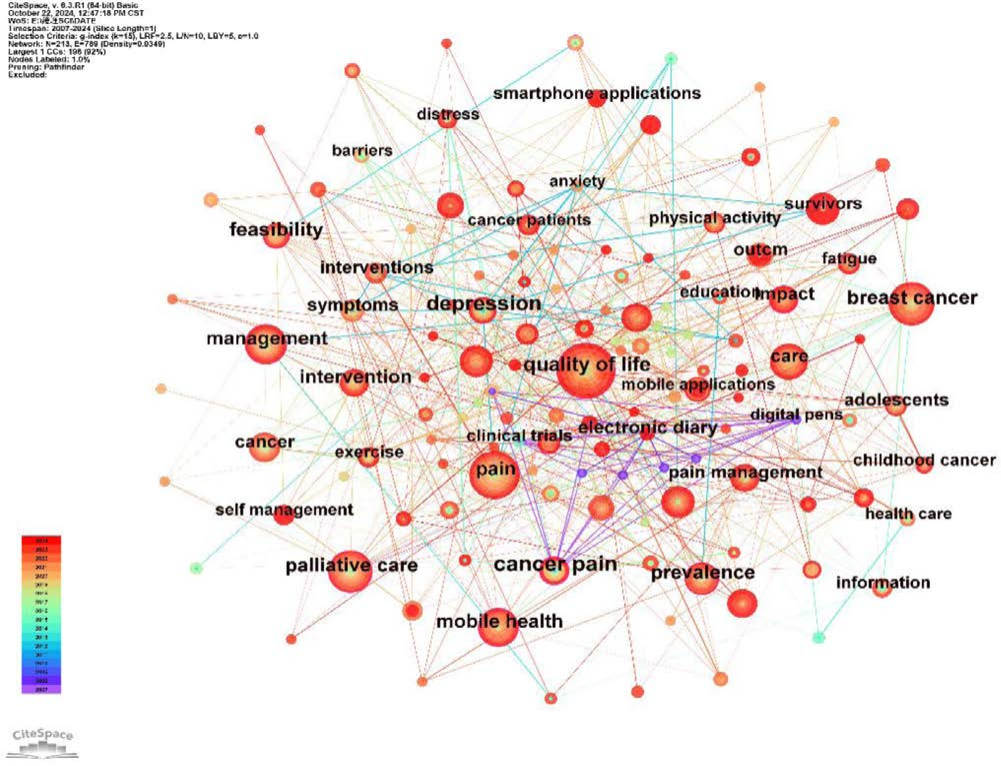
Keyword co-occurrence network. The larger the node is, the higher the frequency of occurrence of this word. There is a purple circle outside the node, indicating that this word is relatively important in the research field.

A total of 213 keywords were retrieved through CiteSpace from the 252 included studies. The words appearing in Figure 7 reflect the forefront of research, and the first 5 high-frequency keywords are quality of life, pain, breast cancer, palliative care, management, and mobile health. The more keywords that appear, the larger the nodes. The word with the highest centrality is quality of life, indicating that the research content represented by this word is relatively important.

**Figure 8.**
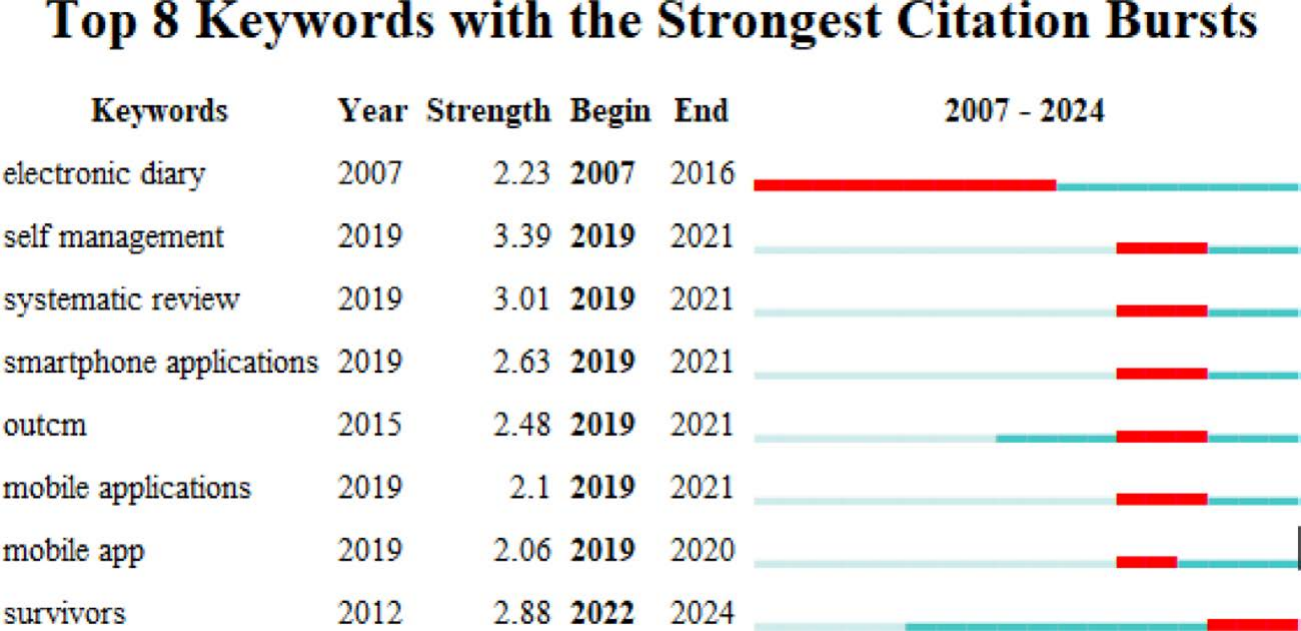
The top 8 keywords with the most prominent citation bursts. The red line in the figure is used to indicate the intensity of the citation burst, and the starting and ending points of the red line correspond to the start and end times of the burst, respectively.

Figure 8 shows that these words appeared between 2007 and 2024, and self-management ranked first with the highest burst intensity (3.39), followed by systematic reviews (3.01) and survivors (2.88). In recent years, keywords such as survivors, mobile apps, outcomes, smartphone applications, and self-management have attracted much attention from researchers in terms of their abrupt ends (Fig. 8).

**Figure 9.**
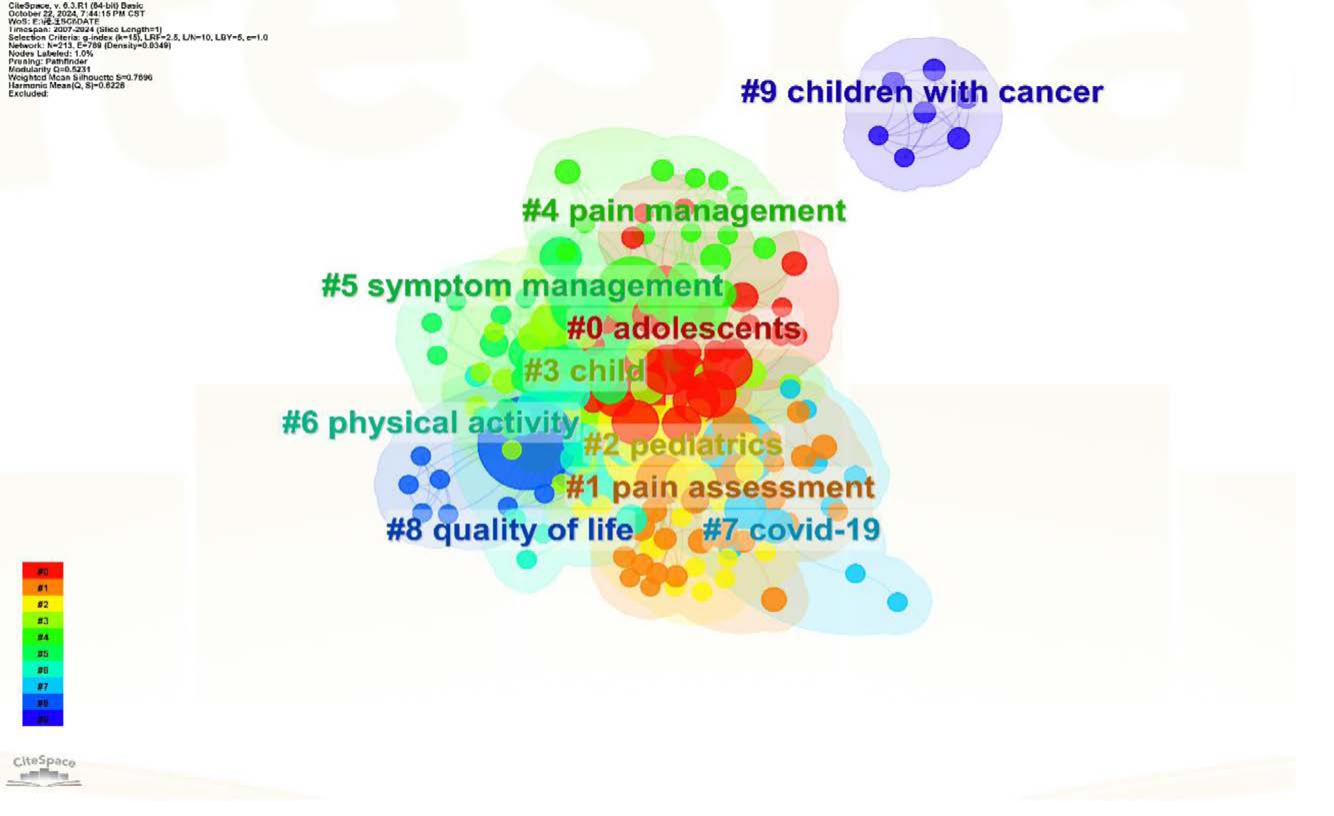
The keyword clustering network. It shown in is characterized by the co-occurrence network clustering of keywords forming irregular regions, with each region having a label from 0 to 9. The smaller the label number is, the more keywords are contained in the cluster. Each cluster is composed of multiple highly correlated keywords.

Figure 9 presents the clustering analysis of keyword co-occurrence from 2007 to 2024, specifically focusing on publications related to mobile apps for managing cancer pain. This analysis involved a total of 213 keywords. The studies were grouped based on the degree of proximity among the keywords, resulting in 10 clusters numbered from 0 to 9 (Fig. 9). Notably, a lower cluster number indicates a higher concentration of keywords within that cluster.

**Figure 10.**
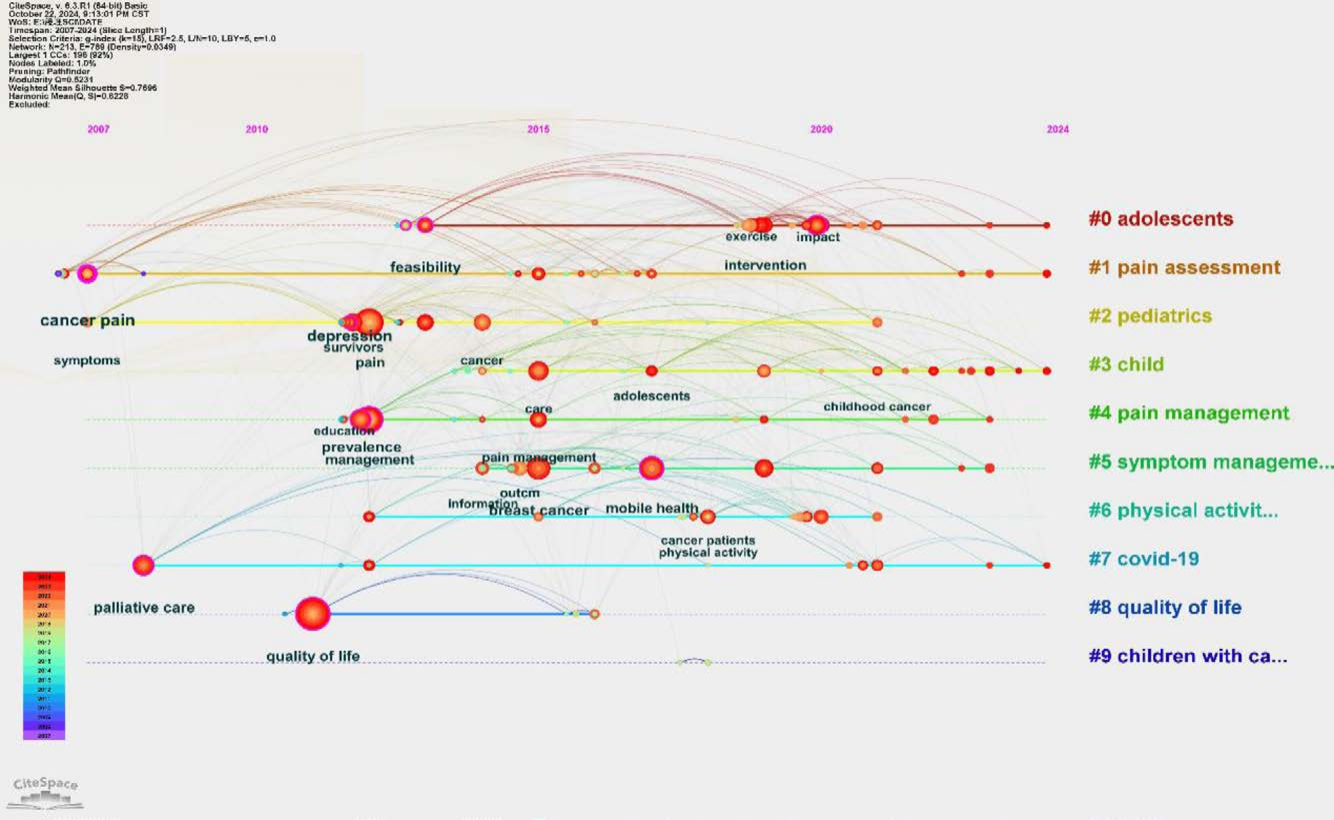
The timeline view of the keyword clusters. The node size reflects the frequency of keyword citations, whereas the lines connecting nodes indicate the co-occurrence of keywords.

Figure 10 visually displays the associations between keywords at various time points using a timeline view. From this, we can observe that “adolescents” and “pain assessment” are currently the 2 most extensively researched areas.

## 4. Discussion

This study employed bibliometric methods to analyze 252 articles focused on the use of mobile app digital healthcare for cancer pain management from 2007 to 2024. The analysis included original research articles (78.6%) and review articles (21.4%). Since 2007, research on the digital management of cancer pain through mobile apps has shown a consistent growth trend, peaking in 2023. This trend clearly shows that the application of mobile app digital healthcare in cancer pain treatment is increasingly recognized and valued. Researchers are increasingly concentrating on and investigating the potential of mobile app digital healthcare to alleviate cancer pain. In terms of research institutions, journal publications, and the overall number of publications, the United States holds a leading position in the field of mobile app digital healthcare for cancer pain management.

We analyzed the journals with the highest citation frequency, identifying the top 5 as Supportive Care in Cancer, Journal of Clinical Oncology, Journal of Medical Internet Research, Journal of Pain and Symptom Management, and JMIR mHealth and uHealth. All of these journals are based in the United States, underscoring the country’s prominent position in this field. Further analysis of the research focus of these journals revealed that pain management, cancer treatment, and mobile application development are the predominant research directions. Consequently, research on the use of mobile applications for digital healthcare in managing cancer pain is continually expanding and evolving.

**Figure 11.**
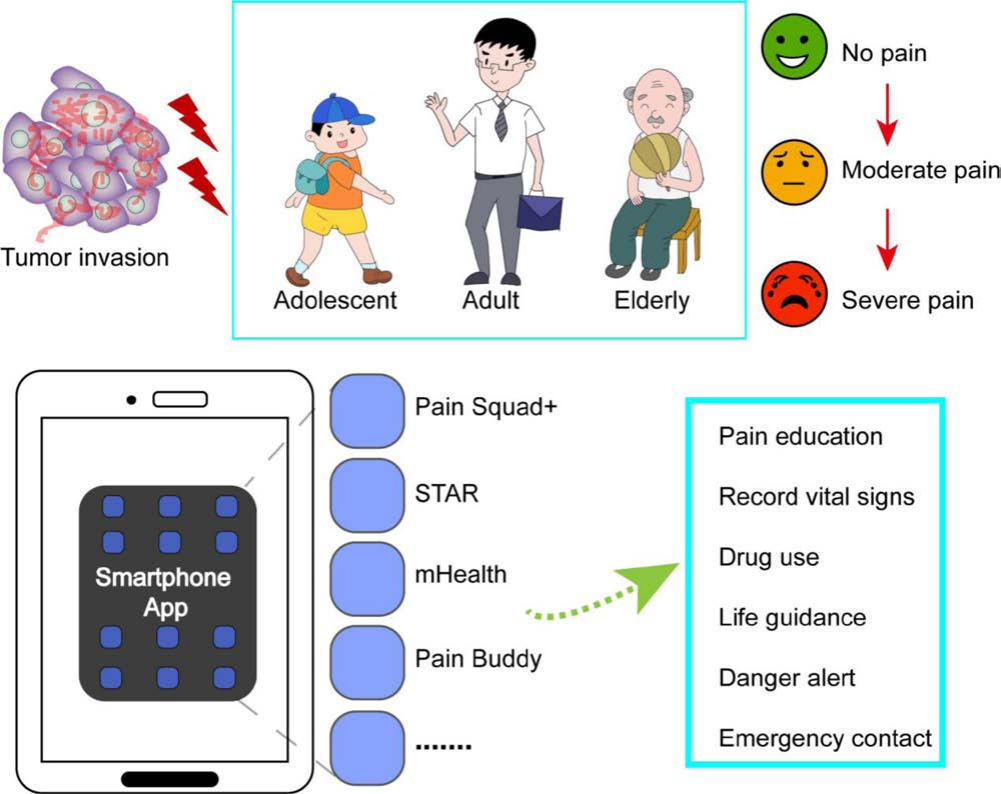
The main mechanism of digital healthcare control for cancer in mobile apps.

Table 2 lists the 9 most cited studies, including 6 research articles, 2 systematic reviews, and 1 meta-analysis, which we discuss below. Fortier MA et al^[16]^ reported the highest total citation count (17 times) for their papers published in 2016. The traditional treatment model has certain shortcomings. Advanced cancer patients cannot seek medical treatment face-to-face, and opioid drugs have various side effects. Mobile apps are auxiliary treatments that combine the application of opioid drugs with patients’ pain cognition and behavior, which can effectively control cancer pain in patients.^[25]^ Fortier MA et al^[16]^ confirmed this point. Silva et al^[17]^ concluded that mobile apps not only improve the pain symptoms of cancer survivors but also effectively alleviate their fatigue symptoms. Yang et al^[18]^ proposed that the use of multidimensional mobile applications, which have traditionally been unattainable, can monitor patients’ pain symptoms in real time, evaluate the side effects of medication, conduct real-time consultations, and provide effective intervention measures in a timely manner. A survey of teenagers with cancer revealed a desire to develop practical skills for real-time management of disease symptoms.^[26]^ Jibb LA et al^[19]^ developed the mHealth Pain Squad + real-time pain self-management app for adolescent cancer patients, which can calculate pain scores through pain questionnaires filled out by patients to implement strategies for adjusting treatment. Zheng CY et al^[22]^ concluded that mobile apps with instant messaging modules can reduce cancer pain scores. As distraction tools, mobile apps can shift patients’ pain,^[27]^ which is a simple and effective intervention measure.^[28]^ It can not only reduce the side effects of chemotherapy and opioid drugs,^[29,30]^ but also prevent the abuse of opioid drugs from causing patient death.^[31]^ Patients also express that they do not want to use a single opioid drug to manage cancer pain and hope to use mobile apps containing pharmacology, psychological, and behavioral interventions, education, communication, etc, for intervention.^[25]^ The effectiveness of mobile apps as pain management tools.^[32]^ Its functions are diverse, including real-time monitoring, regulation, education, tracking, etc, and it is highly trusted by patients.^[33]^ However, studies have indicated that mobile app digital healthcare can impose economic burdens and increase the degree of complexity of its application for patients.^[34,35]^ Therefore, in the development of mobile APP digital medical applications, many factors need to be considered to solve special problems for different cancer pain patients and provide evidence-based interventions for sustainable monitoring.^[17]^ The main mechanism of mobile app digital medical control for cancer pain is shown in Figure 11.

Through keyword analysis, we observed that quality of life, pain, breast cancer, palliative care, and mobile health are current research hotspots. Among patients with different types of cancer pain, the first concern is their own quality of life, followed by pain. They hope to use opioid drugs while improving their quality of life, but some patients refuse to use opioid drugs as a means of controlling pain, as they feel that they are facing death before using opioid drugs.^[36]^ Therefore, when treating the symptoms of cancer patients, multiple factors, such as physical, psychological, and social factors, should also be considered.^[37]^ Sustainable monitoring and improvement of patients’ physical and psychological conditions, socioeconomic burdens, and other issues can be achieved through mobile app digital healthcare.^[38]^ Breast cancer is one of the most common cancers among women.^[39]^ Seventy-three percent of breast cancer patients can be successfully treated, and the survival period is >5 years.^[40]^ For cancer survivors, these tools are more helpful in improving their quality of life.^[41]^ Therefore, mobile apps for digital healthcare demonstrate enormous potential and can provide valuable therapeutic tools to address this challenge.

## 5. Limitations

Although this article provides a comprehensive analysis of the current status of mobile app digital healthcare in managing cancer pain, there are still several limitations that need to be noted. First, although we have made every effort to extensively collect and include all relevant literature, given the wide variety of cancer types, there may still be some relevant literature that has been overlooked and not included in the scope of this study. Second, our search scope is limited to the WOS core collection; therefore, any papers that may have been overlooked are not included in this core collection. Additionally, while mobile applications encompass a variety of programs, we have not compared the effectiveness of different programs in managing cancer pain. Moreover, we have not conducted a comprehensive analysis to determine which template demonstrates the best performance.

## 6. Prospects

The mechanism of digital medical management for cancer pain through mobile applications, along with research on the therapeutic effects of various programs and modules, is set to become a significant area of research in the future. Given these emerging trends, this study is still in its early stages and requires experimental clinical evidence.

## Acknowledgments

The authors thank Professor Chen Chaomei for generously providing access to CiteSpace.

## Author contributions

**Conceptualization:** Qiu-Song Shen, Hou-Ming Kan, Rui-Yu Wang, Xue-Qin Rong.

**Data curation:** Qiu-Song Shen, Hou-Ming Kan, Nai-Fa Li.

**Formal analysis:** Qiu-Song Shen, Hou-Ming Kan.

**Methodology:** Qiu-Song Shen, Hou-Ming Kan, Rui-Yu Wang.

**Project administration:** Qiu-Song Shen.

**Software:** Rui-Yu Wang.

**Writing – original draft:** Qiu-Song Shen, Hou-Ming Kan.

**Writing – review & editing:** Qiu-Song Shen, Hou-Ming Kan, Sha Wang, Xue-Qin Rong.
